# Sleep quality and its relationship with physical activity and frailty syndrome among hospitalized older adults in North Iran

**DOI:** 10.4314/ahs.v26i1.11

**Published:** 2026-03

**Authors:** Leila Zahed Nakhjiri, Azar Darvishpour, Ali Nouri, Ameneh Beig Mohammadi

**Affiliations:** 1 Department of Nursing, Zeyinab (P.B.U.H) School of Nursing and Midwifery, Guilan University of Medical Sciences, Rasht, Iran; 2 Social Determinants of Health Research Center, Trauma Institute, Guilan University of Medical Sciences, Rasht, Iran

**Keywords:** Aging, Sleep Disorders, Physical Activity, Frailty Syndrome

## Abstract

**Background:**

Sleep disorders are a prevalent concern among older adults, contributing to reduced physical function. A bidirectional relationship between sleep disorders and frailty syndrome has been suggested.

**Objectives:**

This study aimed to investigate sleep quality and its associations with physical activity and frailty syndrome among hospitalized older adults.

**Methods:**

A cross-sectional study was conducted in 2022 among 150 hospitalized older adults in East Guilan, northern Iran, using convenience sampling. PSQI, CHAMPS, and TFI questionnaires were employed as research instruments. Data were analyzed using SPSS version 16, applying descriptive statistics and Spearman correlation coefficients.

**Results:**

The majority (76%) of participants had poor sleep quality, and 58.66% reported being physically active. Additionally, 58% were non-frail. Although no significant relationship was found between the total sleep quality score and frailty syndrome, a significant correlation was observed between habitual sleep efficiency and all dimensions of frailty (physical, psychological, and social). A positive relationship was also found between sleep quality and physical activity (r = 0.20, p = 0.014).

**Conclusion:**

Improved physical activity was associated with better sleep quality, which may reduce risk of frailty. Promoting physical activity and improving sleep hygiene in hospitalized older adults could enhance health outcomes and reduce frailty risk.

## Introduction

Sleep has a profound impact on various aspects of quality of life^1^. The concept of sleep quality is complex and multifaceted, encompassing satisfaction with the sleep experience, including sleep initiation, maintenance, quantity, and refreshment upon awakening^3^. Poor sleep quality is linked to numerous health risks, contributing to poor health outcomes^4^. Sleep disorders, such as insomnia, narcolepsy, and excessive sleepiness, have significant adverse health effects and are prevalent worldwide^5^. Studies have reported high prevalence rates of sleep disorders, including obstructive sleep apnea (41%), hypersomnia (32%), periodic limb movement disorder (30%), and REM sleep behavior disorder (64%)^6^. In China, approximately 38.9% of the population suffers from insomnia^2^,^7^, while a systematic review revealed a 44% prevalence of sleep apnea in Iran^8^. Globally, 30-45% of the population experiences insomnia, with this rate increasing with age^9^.

Aging is associated with changes in sleep quality and structure, leading to sleep disorders and frequent complaints^10^. Sleep disorders are prevalent among older adults, with approximately 50-60% experiencing at least one type of sleep disorder, such as sleep apnea, restless legs syndrome, REM sleep behavior disorder, etc.^11^. Aging causes alterations in both the amount and quality of sleep^12^. The most common sleep disorders in older adults are insomnia, circadian rhythm sleep-wake disorders, excessive daytime sleepiness, sleep-related movement disorders, and sleep-disordered breathing^9^,^13^-^14^. Poor sleep quality is associated with serious negative consequences on physical, mental, and social aspects of well-being^15^. Consequences of poor sleep quality include physical complications (weakness and fatigue, daytime sleepiness, increased risk of falling, slow responses, reduced physical performance)^16^-^18^, psychological complications (irritability, and increased caffeine/alcohol consumption)^16^, and cognitive complications (reduced concentration and decreased cognitive function)^17^,^18^.

In a social-ecological model, three levels of factors play roles in sleep: individual, social, and societal factors^19^. In a review study, the adverse factors affecting the sleep quality of older adults were reviewed, and the results were presented in three domains: person, environment, and occupation^20^. It seems that changes in sleep reflect the natural processes of development, which can be caused by primary factors such as aging, the most important factor in the occurrence of sleep disorders in old age^10^, or secondary factors such as medical or psychiatric diseases^21^. Sleep disorders can also be caused by side effects of drugs and psychosocial factors^22^,^23^. The role of physical activity is especially significant, as it has been shown to improve muscle strength, balance, cognition, depression, and self-efficacy, all of which result in improved functional independence^24^. Physical activity increases energy consumption, improves sleep quality by secreting endocrine hormones, and reduces visceral fat, thereby enhancing sleep depth^20^. Aging is associated with physical inactivity, leading to declines in muscle mass and strength^25^. In older adults with reduced musculoskeletal function, 44-66% report sleep disorders, and 12-28% experience daily drowsiness^26^. A systematic review showed that moderate-intensity physical activity promotes sleep quality in older adults^27^.

Sleep alterations, including poor sleep quality and prolonged latency, are related to frailty in older adults^12^. Frailty is a definite sign of aging and is associated with numerous negative conditions and consequences^28^. The notion of frailty one usually understands as a syndrome of weakness, fragility, or exhaustion of reserves^29^. Although the term “frailty” is the core of geriatrics and gerontology, there is no clear consensus regarding the definition of this condition^30^. Frailty is a complex combination of the natural aging process and various medical problems, characterized by weakness, fragility, or exhaustion of reserves^28^. This syndrome shares several clinical characteristics with sleep-wake cycle disorders, especially excessive daytime sleepiness, with low physical activity levels. The sensation of frailty-related fatigue also shares characteristics of self-reported poor sleep quality^12^. The literature showed a bidirectional relationship between frailty and sleep quality in older adults^31^. Previous studies suggest that poor sleep quality or abnormal sleep duration may be linked to frailty^12^,^32^. Sun et al. found that poor sleep quality, long sleep latency, sleep disturbance, daytime dysfunction, and long sleep duration were associated with increased odds of frailty in older adults^32^. Nóbrega et al. conducted a study investigating the relationship between sleep qualitynd frailty syndrome in older adults, and they found that decreased nighttime sleep duration and delayed sleep apnea were associated with frailty syndrome in older adults^12^. However, more evidence needs to be accumulated before sleep disturbances can be established as a behavioral risk factor for frailty^32^.

Despite existing studies, limited research addresses the relationship between sleep quality, physical activity, and frailty syndrome among hospitalized older adults. Understanding this relationship is crucial, especially in the context of aging populations. It could help counteract poor sleep quality, which is a risk factor for age-related physiological and cognitive decline^33^. Given the increasing number of older adults in Iran, coupled with cultural and social differences, research in this area is essential. In particular, there is limited information regarding the sleep quality of older adults in Guilan, the oldest province in Iran. Recognizing these issues is vital for planning effective interventions and prevention programs. Nurses play a crucial role in assisting patients with sleep disorders and improving sleep quality, which is closely linked to overall health outcomes^16^. This study aimed to assess the sleep quality of older adults and its relationship with physical activity and frailty syndrome, providing valuable insights for improving the health and well-being of hospitalized older adults.

The main research question was:

What is the status of sleep, physical activities, and frailty syndrome among hospitalized older adults?

The hypotheses of the study were:

There is a significant correlation between sleep quality and physical activities among hospitalized older adults.
There is a significant correlation between sleep quality and frailty syndrome among hospitalized older adults.

## Methods

### Study design, setting, and participants

This cross-sectional study was conducted on 150 older adults from June to December 2022. The research setting included public hospitals in East Guilan, northern Iran, from which three hospitals were randomly selected. The inclusion criteria included being 60 years and older, ability to understand and speak Persian, not having cognitive problems (getting a score of more than 8 in the AMT-10), not having communication problems, a history of falling while moving, a history of amputation and disability, and the use of central nervous system drugs during the study. Exclusion criteria included the withdrawal of participants from continuing to cooperate in the study. Convenience sampling was performed based on the inclusion criteria.

To determine the sample size, according to the study of Nóbrega et al. (2014)^12^, the coefficient of determination (r^2^) between sleep status and frailty syndrome was equal to 0.08, the correlation between variables (0.28) and consequently, the confidence interval of correlation coefficient (ω) was calculated to be 0.288. The sample size with a 95% confidence interval and 90% test power was calculated using the formula below, and it was determined to be 135 people. Considering the sample attrition rate (10%), 150 people were finally considered.

### Data collection instruments

Research tools include the Abbreviated Mental Test (AMT), the Pittsburgh Sleep Quality Index (PSQI), the Community Healthy Activities Model Program for Seniors (CHAMPS), and the Tilburg Frailty Index (TFI), which are described below. In the present study, demographic characteristics included age, sex, place of living, level of education, job, history of underlying diseases, use of assistive devices, inpatient ward, and number of drugs used.

### Abbreviated Mental Test (AMT)

This tool comprises 10 simple and concise questions designed to evaluate orientation, concentration, attention, as well as short-term and long-term memory^34^. In this questionnaire, one point is assigned to each correct answer, and the total score is 10 points. The scoring is straightforward, with higher scores indicating better cognitive function. The psychometric properties of the Persian version of this test have been assessed by Bakhtiyari et al. (2014) in Iranian older people. The results indicated that this tool demonstrated good discriminant validity in diagnosing normal and impaired cognitive participants. A cut-off point of AMT < 8 was established, exhibiting a sensitivity of 92.15% and a specificity of 81.50%^35^.

Pittsburgh Sleep Quality Index (PSQI): This questionnaire includes 19 items on 7 components (subjective sleep quality, sleep latency, sleep duration, habitual sleep efficiency, sleep disturbances, use of sleep medication, and daytime dysfunction). These items are rated in terms of the frequency or severity of the problem on a four-point Likert scale (e.g., 0 = Not during the past month, 1 = Less than once a week, 2 = Once or twice a week, 3 = Three or more times a week). The questionnaire's total score ranges from 0 to 21, with higher scores indicating poorer sleep quality. A total score of 6 or more indicates inadequate sleep quality^36^. The psychometric properties of the Persian version of this questionnaire were assessed by Farrahi Moghaddam et al. (2012), and the Persian Cronbach's alpha for the version was 0.77, demonstrating acceptable reliability^37^.

Community Healthy Activity Model Program for Seniors (CHAMPS): The Physical Activity Assessment Questionnaire, designed by Stewart et al.^38^, consists of 41 questions that assess the activities older individuals have engaged in over the past week, relating to the previous month. The intensity of physical activity is determined using MET (Metabolic Equivalent Test), which estimates metabolic expenditure during physical activity. In this questionnaire, the total MET score for each older person is calculated based on the frequency of activities per week and the number of hours spent on them weekly (separately). If the MET score is 1, it means the person is sedentary; if the score is between 1 and 3, it indicates low-level physical activity; a scorbetween 3 and 6 signifies moderate activity, and a score above 6 indicates high-intensity physical activity. To calculate the intensity, the duration of each activity is multiplied by the time spent on that activity per day or week. The psychometric properties of the Persian version of this tool were evaluated by Sahaf et al. (2014), with the reliability reported to be 0.85 Cronbach's alpha coefficient, indicating high internal consistency^39^.

Tilburg Frailty Index (TFI): This tool, designed by Gobbens et al. (2010), screens older individuals for the presence of frailty^40^. It includes 15 questions in three dimensions: physical function, psychological, and social^28^. The physical dimension comprises eight items: physical health, involuntary weight loss, difficulty walking, difficulty maintaining balance, hearing loss, visual impairment, decreased hand strength, and physical fatigue. The psychological dimension consists of four items: memory status, depression, anxiety, and coping ability. The social dimension includes three items: individual life, social relationships, and social support. In this questionnaire, 11 items are answered with the words “yes” or “no,” and 4 items with “yes,” “sometimes,” or “no.” The total score ranges from 0 to 15, and a score of <5 indicates the presence of frailty. The psychometric validation was conducted using a test-retest method, and the reliability was reported to be 0.79^40^. In Iran, the validity of the TFI was assessed by Mazoochi et al. (2020), revealing a sensitivity of 0.95, specificity of 0.86, and an accuracy of 0.88 at the cut-off point of 4.5. The positive and negative predictive values were 0.68 and 0.98, respectively, confirming the instrument's high validity^41^.

### Procedures

To collect data, the researcher introduced herself and explained the research objectives to the patients and obtained informed consent. The researcher then read the questionnaire items to the patients, and the answers were marked in the relevant sections. It took approximately 30 minutes for each patient to complete the questionnaires. The sampling lasted 3 months.

The medical information of all subjects was obtained from medical records and through interviews with the patients. In the present study, the medical record was used to complete the demographic characteristics described above, and the patient's demographic characteristics recorded in the medical record were checked at the same time as asking the patient. This was done to overcome recall bias. Recall bias is a result of a recall error. Occasionally, study participants can erroneously provide responses that depend on his/her ability to recall past events. It's challenging to overcome recall bias in practice. To do so, it's crucial to identify situations where recall errors are more likely to happen. Recall bias has been associated with various factors such as the length of the recall period, the characteristics of the disease being studied, patient/sample characteristics, and study design^42^.

### Data Analysis

The data was analyzed using SPSS software version 16 (IBM Corp., Armonk, NY, USA). Descriptive statistics (to determine the number, percentage, mean, and standard deviation) and Spearman correlation coefficient were used to evaluate the correlation between variables due to the lack of normality. Normality was measured by the Kolmogorov-Smirnov test. All calculations taking into account the level of significance (P <0.05) were performed.

## Ethical considerations

This study was conducted according to the Helsinki Declaration, and the study protocol was approved by the ethics committee of the Vice Chancellor for Research and Technology of Guilan University of Medical Sciences (with the ethics ID IR. GUMS. REC. 1399.326). Before completing the informed consent form, participants were informed of the objectives, implementation method, benefits and potential risks, and the tools used.

## Results

The results of the study showed that most of the participants were in the age group of 60-74 years (59.3%), were female (53.3%), and were married (75.3%). In terms of education, the majority of participants (64%) were illiterate, and most of them (56%) lived in city (Table 1). The results of the study showed that the majority of participants (76%) had poor sleep quality, and more than half of the older adults (58.66%) were physically active. Additionally, more than half of the older adults (58%) did not have frailty syndrome. The sleep quality, physical activity, and frailty syndrome status of the participants are presented in Table 2.

**Table 1:** Demographic characteristics of hospitalized older adults (n = 150)

%	n	variable
		**Age (years)**
59.3	89	60 – 74
40.7	61	75 - 85
		**Sex**
46.7	70	Men
53.3	80	Female
		**Marital status**
3.3	5	Single
75.3	113	Married
21.3	32	Widow
		**Place of living**
56	84	City
44	66	Village
64	96	**Level of education**
		Illiterate
26	39	High school
6	9	Diploma
4	6	University
		**Job**
40.7	61	Housewife
6.7	10	Freelance job
18.7	28	Retired
23.3	35	Farmer
9.3	14	Disabled
1.3	2	Unemployed
		**History of underlying diseases**
25.3	38	Visual disorder
12.7	19	Hearing disorder
13.3	20	Thyroid disorder
37.3	56	Diabetes
50.7	76	Cardiovascular disease
44.7	67	Hyperlipidemia
74	111	High blood pressure
11.3	17	Nervous disorder
26.7	40	Skeletal disorder
		**Inpatient ward**
6.67	10	Emergency
42.67	64	Internal
20	30	Surgery
30.66	46	CCU – post CCU
		**Using assistive devices**
27.3	41	Mobility assistive device
41.3	62	visual assistive device
8.7	13	hearing assistive device
		**Number of drugs used**
66	99	Less than 5
34	51	≥ 5

Note: SD (Standard Deviation), Min (Minimum), and Max (Maximum)

**Table 2:** Sleep quality, physical activity, and frailty syndrome status of the hospitalized older adults (n = 150)

variable	number	percent	Mean±SD	Min	Max
**Sleep quality**					
Optimal sleep quality	36	34	4±1.171	1	5
Poor sleep quality	114	76	11.17±3.917	6	21
**Physical activity**					
Immobile	3	2	70.0±75.498	0	150
Low mobility	5	4	522.60±83.149	378	585
Moderate mobility	53	35.23	1601.48±536.434	690	2702
Active	88	58.66	8753.65±4999.534	3036	37031
**Frailty syndrome**					
Frail	60	42	6.94±1.831	5	12
Non-frail	87	58	2.97±0.958	1	4

Note: SD (Standard Deviation), Min (Minimum), and Max (Maximum)

The mean scores of sleep quality, frailty syndrome, and their subscales among older adults are shown in Table 3.

**Table 3:** The mean score of sleep quality, Frailty syndrome, and their subscales among hospitalized older adults (n=150)

Variable	Subscales	Mean	SD	Min	Max
Sleep quality	Subjective Sleep Quality	20.50	5.08	0	24
	Sleep Latency	52.30	39.19	10	180
	Sleep Duration	7.35	3.23	2	30
	Habitual Sleep Efficiency	6.06	1.29	2	10
	Sleep Disturbances	1.29	0.72	1	4
	Use of Sleep Medication	1.85	1.30	1	4
	Daytime dysfunction	2.39	1.08	1	4
	Total PSQI score	9.09	4.35	1	21
Frailty syndrome	Physical	2.44	1.83	0	7
	Physiological	1.33	0.96	0	5
	Social	0.95	0.56	0	3
	Total Frailty syndrome score	4.63	2.40	1	13

Note: SD (Standard Deviation), Min (Minimum), and Max (Maximum)

The findings of this study revealed that the total sleep quality was not significantly related to frailty syndrome, as indicated by the Spearman correlation coefficient test (r = 0.007, p = 0.930). However, a significant relationship was found between habitual sleep efficiency and all dimensions of frailty syndrome (physical, psychological, and social), with p-values of 0.006, 0.004, and 0.044, respectively. Furthermore, subjective sleep quality, sleep latency, and daytime dysfunction were significantly related to the physical dimension of frailty syndrome, with p-values of 0.001, 0.023, and 0.017, respectively. Moreover, a significant relationship was found between sleep duration and the social dimension of the frailty syndrome based on the Spearman correlation coefficient test (r = -0.162, p = 0.047). The results also indicate a significant correlation between sleep quality and physical activity in older adults (r = 0.20, p = 0.014) (Table 4).

**Table 4:** Correlation between sleep quality with frailty syndrome and physical activity among hospitalized older adults (n = 150)

								Dimensions of sleep quality								
															Total sleep quality	
variable	Subjective sleep quality		Sleep latency		Sleep duration		Habitual sleep efficiency		Sleep disturbances		Use of sleep medication		Daytime dysfunction			
	r	p	r	p	r	p	r	p	r	p	r	p	r	p	r	p
**Dimensions of frailty syndrome**																
Physical	-0.270	0.001**	0.185	0.023*	-0.069	0.400	0.224	0.006**	0.127	0.093	0.129	0.115	0.195	0.017*		
Physiological	-0.057	0.488	0.078	0.144	-0.124	0.0130	0.236	0.004**	0.018	0.825	-0.046	-0.572	0.127	0.121	0.007	0.930
social	-0.191	0.020*	0.117	0.155	-0.162	0.047*	0.164	0.044*	0.102	0.215	-0.041	0.614	-0.050	0.546		
**Physical activity**	0.325	0.000**	-0.011	0.889	0.147	0.074	-0.176	0.031*	0.127	0.122	-0.177	0.031*	-0.115	0.162	0.200	0.014*

r: Spearman correlation coefficient; p: significance level

* Correlation is significant at the 0.05 level (2-tailed)

** Correlation is significant at the 0.01 level (2-tailed)

## Discussion

The results regarding the sleep status of the participants revealed that the majority (76%) experienced poor sleep quality. This finding is supported by studies conducted by Kulpatcharapong et al. (2020), who reported a high prevalence of poor sleep quality among hospitalized patients after the first night of admission, comparing baseline sleep quality at home (50% vs. 18.8%)^43^. Similarly, Azri et al. (2016) in Brazil also indicated that most older adults experienced poor sleep quality^44^. The findings of the present study align with those of previous research. Along with increasing age, major changes in sleep patterns and quality occur, leading to sleep disorders and recurrent complaints^45^. On the other hand, hospitalization is a period of acute sleep deprivation for older adults due to environmental, medical, and patient factors. Although hospitalized patients need adequate rest and recovery during acute illness, older patients face unique risks due to acute sleep loss during hospitalization^46^. In a study, almost half (49.2%) of the patients tested for insomnia^47^. Another study demonstrated poor sleep quality and quantity of inpatients and a drastic decrease in sleep quality in the hospital versus at home^48^. In contrast, a study conducted in China found that 66.3% of older adults had adequate sleep^49^, which contradicts our findings. This discrepancy may be attributed to cultural and lifestyle differences between the populations and differences in the research settings of the two studies.

In this study, the majority of older adults were found to have sufficient levels of physical activity, with over half of them (58.66%) being active and 35.33% having moderate mobility. This discovery aligns with the findings of studies by Van Dyck et al. (2016)^50^ and Menai et al. (2014)^51^. The high level of mobility observed in the older adults in our study can be attributed to their lifestyle and involvement in agriculture, farming, and horticulture, as well as the favorable climate that is conducive to these activities.

Findings from this study showed that more than half of older adults (58%) do not have frailty syndrome. This result is consistent with the study by Van Oostrom et al.^52^, in which only about 17% of the study population had frailty syndrome in one or more areas. They showed that regular physical activity was associated with a lower risk of frailty in all areas. In addition, considering that in the current study, more than half of older adults were active, and by considering the relationship between continuous physical activity and reducing the risk of frailty syndrome, the results of the present study can be justified. In contrast, a study by Sun et al. (2020) found that 62% of older adults have frailty syndrome or pre-frailty syndrome^32^, and in the study of Nóbrega et al. (2014), 94.3% of older adults had frailty and pre-frailty syndrome^12^, which shows that the present study is inconsistent with these two studies. This result can be due to the lower average age in the present study than in the previous two studies. Aging is one of the most important factors of frailty syndrome, which, due to changes that occur naturally and due to some diseases in older adults, exposes them to frailty syndrome^53^.

The study's results demonstrated a link between sleep quality and physical activity, indicating that enhancing physical activity among older adults improves their sleep quality. This finding is consistent with the results of studies by Štefan et al. (2018)^54^, and Garfield et al. (2016)^55^. In a study conducted by Lorber et al. (2023), it was found that older adults, whether living in the community or a nursing home, can improve their sleep quality and prevent frailty by being physically active^56^. Another study revealed that moderate physical activity is more effective than intensive activity in improving sleep quality for older adults^57^. Additionally, a systematic review demonstrated that physical activity plays a role in preventing frailty^58^.

Furthermore, the findings of a study by Gothe et al. indicate a significant correlation between physical activity and sleep quality, with sleep quality being an important factor in the overall quality of life^59^.

Interrupted sleep and reduced REM sleep duration disrupt the production of sex hormones that are important for muscle production. A decrease in these hormones leads to a decrease in muscle mass and strength^60^. Therefore, those who get good quality sleep can benefit from increased levels of certain hormones, leading to better muscle strength and higher levels of physical activity. Conversely, individuals who are physically active during the day and exposed to sunlight can regulate and improve their body's 24-hour cycle and sleep hormones, ultimately improving the quality of sleep^61^.

The results of this study also indicated that older adults who get sufficient sleep are less likely to develop frailty syndrome. This finding is consistent with the results of studies by Baniak et al. (2020)^62^, and Van Oostrom et al. (2017)^52^. In a systematic review, there was evidence for an association between perceived sleep quality and frailty among older adults^63^. Similarly, a systematic review and meta-analysis showed that longer and shorter sleep durations are associated with an increased risk of frailty^64^. In the present study, sleep duration was significantly associated with the social dimension of frailty syndrome. By controlling and following the physical activities of older adults in various ways, it is possible to prevent the occurrence of sleep disorders and frailty syndrome caused by sleep problems and to provide the ground for promoting the health of older adults and improving their quality of life.

The researchers hope that the study findings will provide valuable information to health policymakers, medical staff, patients, and families, enabling effective measures to enhance the well-being of older adults. Health managers and policymakers should consider planning to develop interventions to encourage physical activity, increase sleep quality, and reduce frailty. The results of this research can help health policymakers formulate strategies to enhance sleep quality among older persons, particularly in hospitals where sleep quality is considerably poorer. Health managers can add measuring the sleep quality of older adults to the fixed and continuous implementation of the protocol in various hospital wards and health centers to reduce the possibility of mobility disability in older adults through monitoring and improving the quality of sleep.

## Limitations

The present study has some limitations that that must be acknowledged. The research focused on the quality of sleep, physical function, and frailty syndrome in older adults hospitalized in public hospitals in East Guilan, Iran. As a result, the findings may not be generalizable to older adults in other geographic regions or those living outside the hospital setting. Furthermore, the cross-sectional nature of the study limits the ability to draw causal inferences between sleep quality, physical activity, and frailty syndrome. Recall bias is another potential limitation, as self-report questionnaires were used to assess physical activity and sleep patterns.

## Recommendations for Future Research

Future research could benefit from longitudinal designs that track the long-term effects of physical activity on sleep quality and frailty syndrome in older adults. Additionally, examining the impact of specific types of physical activities, such as aerobic, resistance training, or a combination, could yield more detailed insights into their respective roles in improving sleep and reducing frailty. Further studies might also investigate the cultural and environmental factors that influence sleep and physical activity patterns in older adults, particularly in diverse settings and populations. Additionally, incorporating objective measures of physical activity and sleep (e.g., wearable devices) would enhance the accuracy of data and the findings' robustness.

## Clinical Implications for Health Managers and Policymakers

The findings of this study suggest that improving sleep quality and encouraging physical activity among older adults can be key strategies for preventing frailty syndrome. Health managers and policymakers should consider implementing programs and interventions aimed at promoting physical activity, particularly in hospital settings where sleep disturbances are more prevalent. Additionally, policies that support the integration of physical activity into the daily routines of older adults, whether through structured programs or community-based initiatives, are critical for improving sleep quality and overall health. The evidence from this study can also inform health policies that focus on improving the quality of life and reducing frailty in the aging population.

## Conclusion

The present study found a significant correlation between sleep quality and physical activity levels among older adults, with those engaging in higher levels of physical activity exhibiting better sleep quality. Additionally, a clear association was observed between habitual sleep efficiency and various dimensions of frailty syndrome. These findings underscore the importance of promoting physical activity as an effective strategy to improve sleep quality and reduce the risk of frailty syndrome in older adults. Given that physical activity plays a crucial role in mitigating the adverse effects of poor sleep, health managers and policymakers should prioritize interventions aimed at increasing physical activity among older populations. Furthermore, ensuring adequate sleep quality in hospitalized older adults is essential to prevent frailty and enhance overall well-being. Future studies should explore the long-term effects of physical activity on sleep and frailty in diverse populations, as well as investigate the mechanisms underlying the relationship between sleep quality and physical health in aging adults.
